# Brain Metastases Unresponsive to Immunotherapy Detected by 18F-FDG-PET/CT in a Patient with Melanoma

**DOI:** 10.3390/diagnostics10060410

**Published:** 2020-06-17

**Authors:** Rosa Fonti, Sara Pellegrino, Ciro Gabriele Mainolfi, Elide Matano, Silvana Del Vecchio

**Affiliations:** 1 Institute of Biostructures and Bioimages, National Research Council, Via Tommaso De Amicis 95, 80145 Naples, Italy; 2 Department of Advanced Biomedical Sciences, University of Naples Federico II, 80131 Naples, Italy; sara.pellegrino@unina.it (S.P.); cirogabriele.mainolfi@unina.it (C.G.M.); delvecc@unina.it (S.D.V.); 3 Department of Clinical Medicine and Surgery, University of Naples Federico II, 80131 Naples, Italy; ematano@unina.it

**Keywords:** 18F-FDG-PET/CT, melanoma, immunotherapy, brain metastases

## Abstract

Recently, newer therapies such as immunotherapy have been increasingly used in the treatment of several tumors, including advanced melanoma. In particular, several studies showed that the combination of ipilimumab, an anti-Cytotoxic T-lymphocyte Associated Protein 4 (CTLA-4) monoclonal antibody and nivolumab, an anti-Programmed Death 1 (PD-1) monoclonal antibody, leads to improved survival in patients with metastatic melanoma. Despite that, immunotherapeutic agents may not reach therapeutic concentration in the brain due to the blood–brain barrier. We report the case of a 50-year-old man with advanced melanoma who underwent whole-body 18F-FDG-PET/CT before and after treatment with immunotherapy showing resistant brain metastases confirmed by subsequent MRI of the brain. Moreover, 18F-FDG-PET/CT was able to detect an immune-related adverse event such as enterocolitis that contributed to the worsening of patient conditions. This case shows how a whole-body methodology such as 18F-FDG-PET/CT can be useful in identifying melanoma cancer patients unresponsive to immunotherapy that may benefit from traditional palliative therapy in the effort to improve their quality of life.

**Figure 1:**
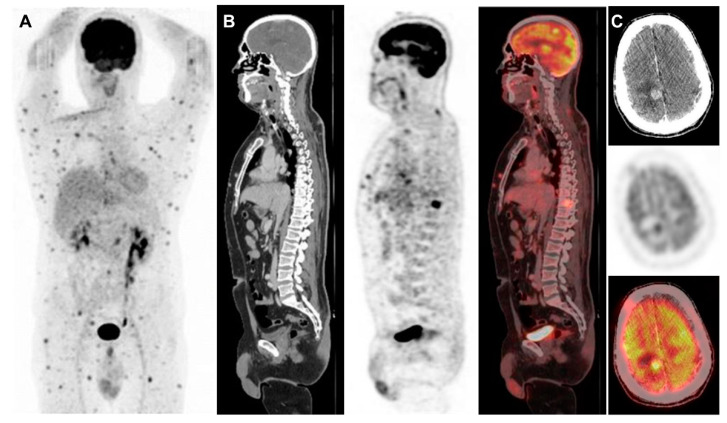
50-Year-old man diagnosed with nodular melanoma, underwent surgical removal of the lesion in the right dorsal region and biopsy of the sentinel lymph node in the right axilla, that showed no metastatic involvement at histopathological examination. After ten months, a new lesion appeared in the right clavicular region. The patient, therefore, underwent local surgery with lymphadenectomy of right cervical lymph nodes that were metastatic at histology with no mutations of BRAF proto-oncogene by polymerase chain reaction. Thus, the patient could not benefit from therapy with BRAF inhibitors targeting the protein kinase BRAF. This kinase, when mutated, drives neoplastic transformation through the constitutive activation of the downstream signaling pathway regulating cell division and differentiation [1]. Whole-body 18F-FDG-PET/CT and contrast-enhanced CT of the brain, performed after surgery, showed no evidence of disease. In the following six months, multiple subcutaneous nodules were observed throughout the body and therefore, a 18F-FDG-PET/CT scan was performed. (**A**) Maximum intensity projection view; (**B**) Sagittal whole-body CT, PET and fusion images; (**C**) Transaxial CT, PET and fusion images of the brain. The scan showed multiple sites of metastatic involvement in various districts throughout the body. In particular, focal 18F-FDG uptake was found in a brain lesion of the right parietal lobe; in a lytic lesion of the frontal bone (SUVmax 7.1); in multiple cervical, thoracic and abdomino-pelvic lymph nodes (SUVmax 6.3); in a lytic lesion of the tenth thoracic vertebra (SUVmax 8) and in multiple nodular lesions in the subcutaneous and muscular tissues throughout the body (SUVmax 8.2).

**Figure 2:**
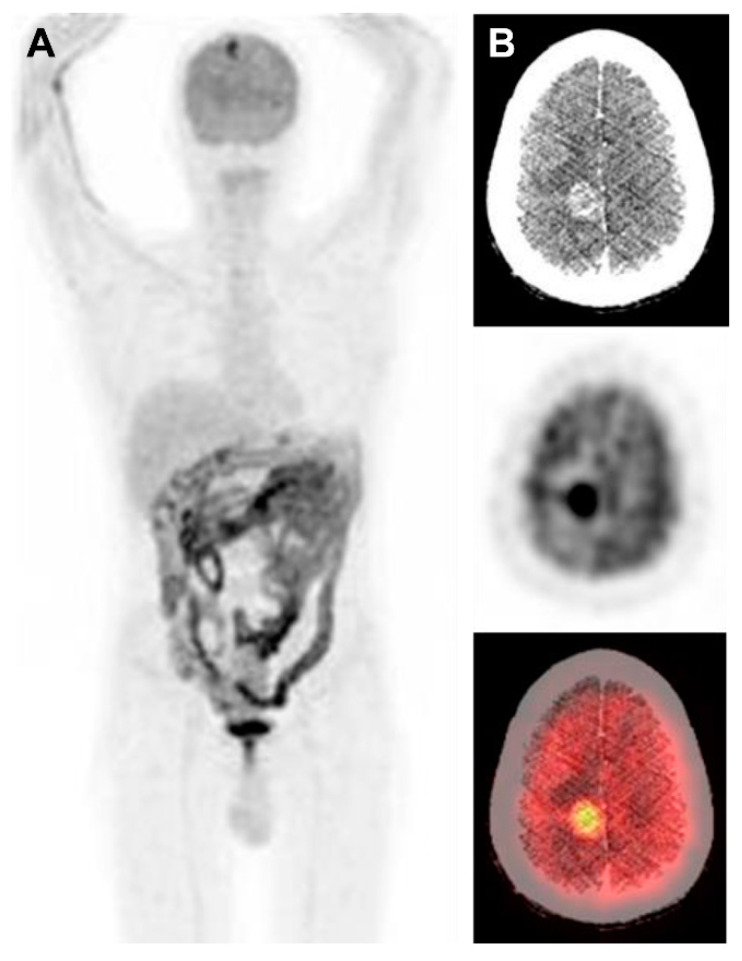
Patient showed no neurologic symptoms nor had been previously treated with steroids, therefore, underwent immunotherapy with ipilimumab in combination with nivolumab [2,3]. During immunotherapy, a reduction of the number and volume of subcutaneous nodules was clinically observed while the patient showed diarrhea, abdominal pain and weight loss likely due to immune-related adverse effects of combined immunotherapy [4]. After the administration of four cycles of immunotherapy with ipilimumab plus nivolumab, a whole-body 18F-FDG-PET/CT scan was performed to evaluate treatment response [5,6]. (**A**) Maximum intensity projection view; (**B**) Transaxial CT, PET and fusion images of the brain. The study showed no pathological 18F-FDG uptake in all previously observed sites of disease, with the exception of brain metastases. In fact, the brain lesion in the right parietal lobe, already visible in the pre-immunotherapy 18F-FDG-PET/CT, showed an increase in both size and 18F-FDG uptake, and other smaller brain metastases were detected. Furthermore, intense and diffuse 18F-FDG uptake was visible in the bowel likely due to enterocolitis induced by immunotherapy [5,7]. Based on 18F-FDG-PET/CT results, MRI of the brain was prescribed [8].

**Figure 3:**
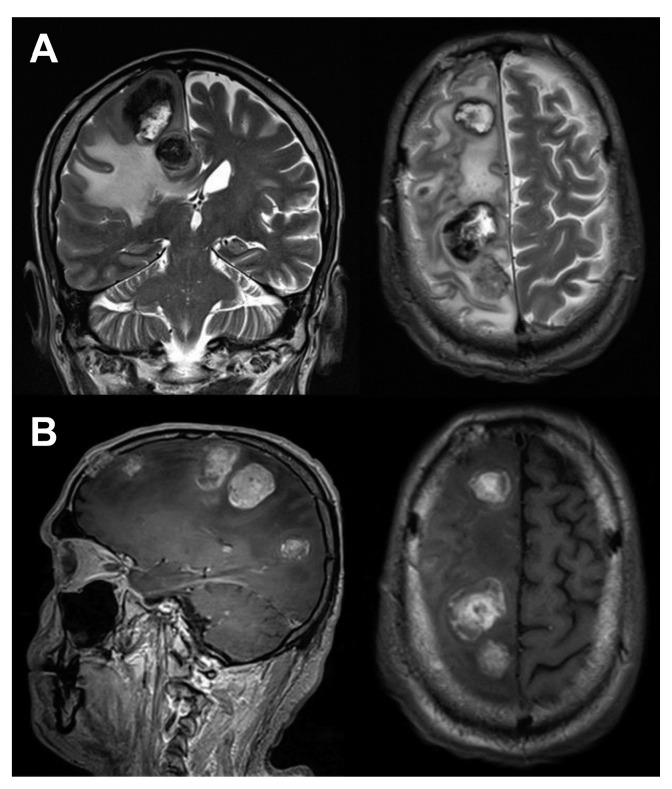
MRI of the brain performed subsequently to 18F-FDG-PET/CT. (**A**) Coronal and transaxial T2 images; (**B**) Sagittal and transaxial T1 TSE images. The study showed multiple metastatic lesions, widespread throughout the brain, hypointense on T2 and hyperintense on T1 images.Moreover, a large edema in the white matter of the right cerebral hemisphere and a lesion in the frontal bone infiltrating the meninx were observed. Therefore, due to extensive brain metastases [9,10,11,12] and worsening of treatment adverse effects [4], immunotherapy was discontinued and the patient was subjected to the best supportive care until his conditions progressively worsened to death after a few months.
